# Patterns of Mesenchymal Condensation in a Multiscale, Discrete Stochastic Model

**DOI:** 10.1371/journal.pcbi.0030076

**Published:** 2007-04-27

**Authors:** Scott Christley, Mark S Alber, Stuart A Newman

**Affiliations:** 1 Department of Computer Science, University of Notre Dame, Notre Dame, Indiana, United States of America; 2 Interdisciplinary Center for the Study of Biocomplexity, University of Notre Dame, Notre Dame, Indiana, United States of America; 3 Department of Mathematics, University of Notre Dame, Notre Dame, Indiana, United States of America; 4 Department of Cell Biology and Anatomy, New York Medical College, Valhalla, New York, United States of America; University of Auckland, New Zealand

## Abstract

Cells of the embryonic vertebrate limb in high-density culture undergo chondrogenic pattern formation, which results in the production of regularly spaced “islands” of cartilage similar to the cartilage primordia of the developing limb skeleton. The first step in this process, in vitro and in vivo, is the generation of “cell condensations,” in which the precartilage cells become more tightly packed at the sites at which cartilage will form. In this paper we describe a discrete, stochastic model for the behavior of limb bud precartilage mesenchymal cells in vitro. The model uses a biologically motivated reaction–diffusion process and cell-matrix adhesion (haptotaxis) as the bases of chondrogenic pattern formation, whereby the biochemically distinct condensing cells, as well as the size, number, and arrangement of the multicellular condensations, are generated in a self-organizing fashion. Improving on an earlier lattice-gas representation of the same process, it is multiscale (i.e., cell and molecular dynamics occur on distinct scales), and the cells are represented as spatially extended objects that can change their shape. The authors calibrate the model using experimental data and study sensitivity to changes in key parameters. The simulations have disclosed two distinct dynamic regimes for pattern self-organization involving transient or stationary inductive patterns of morphogens. The authors discuss these modes of pattern formation in relation to available experimental evidence for the in vitro system, as well as their implications for understanding limb skeletal patterning during embryonic development.

## Author Summary

The development of an organism from embryo to adult includes processes of pattern formation that involve the interactions over space and time of independent cells to form multicellular structures. Computational models permit exploration of possible alternative mechanisms that reproduce biological patterns and thereby provide hypotheses for empirical testing. In this article, we describe a biologically motivated discrete stochastic model that shows that the patterns of spots and stripes of tightly packed cells observed in cultures derived from the embryonic vertebrate limb can occur by a mechanism that uses only cell–cell signaling via diffusible molecules (morphogens) and cell substratum adhesion (haptotaxis). Moreover, similar-looking patterns can arise both from stable stationary dynamics and unstable transient dynamics of the same underlying core molecular–genetic mechanism. Simulations also show that spot and stripe patterns (which also correspond to the nodules and bars of the developing limb skeleton in vivo) are close in parameter space and can be generated in multiple ways with single-parameter variations. An important implication is that some developmental processes do not require a strict progression from one stable dynamic regime to another, but can occur by a succession of transient dynamic regimes tuned (e.g., by natural selection) to achieve a particular morphological outcome.

## Introduction

**Figure 1:**
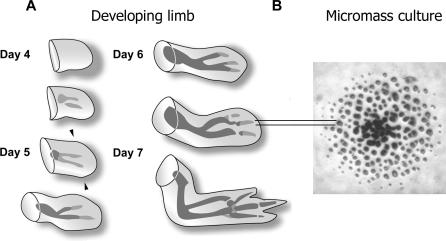
Developing Limb and Micromass Culture (A) Progress of limb skeletal development in chicken forelimb (wing) between 3 and 7 d of embryogenesis. Gray represents precartilage condensation, and black represents definitive cartilage. The developing limb, or limb bud, is paddle-shaped, being flatter in the back-to-front (dorsoventral) dimension than in the thumb-to-little finger (anteroposterior) dimension, or the shoulder-to-fingertips (proximodistal) direction in which it mainly grows. The cartilages that prefigure the bones first arise as stripe-like (e.g., long bones, digits) or spot-like (e.g., wrist bones shown here, or ankle bones in the hindlimb) mesenchymal condensations. The apical zone of the 5-d chicken wing bud (indicated by the arrowheads) or leg bud provide a source of not-yet-condensed mesenchymal cells that when grown in high-density “micromass” culture will form precartilage condensations. (B) Discrete spot-like cartilage nodules that have formed after 6 d in a micromass culture of 5-d leg bud apical zone limb mesenchymal cells, visualized by staining with Alcian blue. The cells in these cultures are initially plated as a densely packed monolayer (“micromass”) and rearrange over short distances in the 2-D plane of the ∼3 mm diameter culture during the indicated period. Each nodule arises from a condensation containing approximately 30–50 cells. As indicated by the parallel lines, the spatial scale of the spot-like nodules (and the precartilage condensations from which they arise) in the micromass cultures is comparable to the diameter of the precartilage and cartilaginous skeletal primordia in the developing limb. The left panel is adapted, with changes, from [54]; the right panel is courtesy of Dr. Sherry Downie.

Skeletal pattern formation in the developing vertebrate limb depends on interactions of precartilage mesenchymal cells with factors that control the spatiotemporal differentiation of cartilage. The most fundamental skeletogenic processes involve the spatial separation of precartilage mesenchyme into chondrogenic and nonchondrogenic domains [1], and can be studied in vitro as well as in vivo (Figure 1). In high-density “micromass” cultures of chondrogenic (i.e., cartilage-forming) embryonic limb mesenchymal cells [2,3], as well as in the developing limb itself [4], morphogens of the TGF-β family induce the local aggregation or condensation of these cells by a process that involves the upregulation of the adhesive extracellular glycoprotein fibronectin [3,5]. Cells first accumulate in regions of increased cell–fibronectin adhesive interactions [6–8] and then acquire epithelioid properties by upregulation of cell–cell adhesion molecules [9,10]. Cartilage differentiation follows at the sites of condensation both in vitro and in vivo (see [11–13] for reviews).

In certain developmental processes, such as angiogenesis (sprouting of capillaries) and invasion by cancer cells of surrounding tissues, pre-existing multicellular structures become more elaborate. Precartilage condensation, by contrast, is an example of a developmental process in which cells that start out as independent entities interact to form multicellular structures. Others in this second category include vasculogenesis (the initial formation of blood vessels), the formation of feather germs, and the aggregation of social amoebae into streams and fruiting bodies. Both continuous [14–18] and discrete [18–29] models have been used previously to analyze a wide range of pattern formation behaviors in both categories using concepts such as chemotaxis, haptotaxis, and reaction–diffusion instability. Discrete models describe the behaviors and interactions of individual biological entities such as organisms, cells, proteins, etc. They are often applied to microscale events where a small number of elements can have a large (and stochastic) impact on a system.

In a previous study [26] we presented a discrete “biological lattice gas” model for high-density cultures of precartilage mesenchymal cells derived from the embryonic vertebrate limb. This model, which was based on the physical notion of a lattice gas, in which individual particles are free to move from point to point on a lattice at discrete time-steps, accurately simulated the formation of patterns of mesenchymal condensations observed in high-density micromass cultures of such cells. In these simulations, the distribution and relative size of the condensations corresponded to in vitro values when appropriate quantities for cell behavioral parameters were chosen, and the simulated patterns were robust against small variations of these values. Moreover, the simulated patterns were altered similarly to the cultures when cell density and exposure to or expression of molecular factors represented in the model were altered in a fashion analogous to their counterparts in the living system.

In the earlier model, each of the limb precartilage mesenchymal cells, and each molecule from a “core” subset of the molecules they secrete (the diffusible activator morphogen TGF-β, a diffusible inhibitor of TGF-β's effects, the extracellular matrix [ECM] protein fibronectin), was represented as a single particle (pixel) on a common grid. Default motion of the cell particles was random, but cell movement was also biased by the presence of fibronectin particles produced and deposited by the cells according to a set of rules involving TGF-β and inhibitor particles. The latter in turn were produced in a cell-dependent fashion according to a reaction–diffusion scheme, the network structure of which was suggested by in vitro experiments [2,3,30].

The ability of the model of Kiskowski et al. [26] to simulate both qualitative and quantitative aspects of precartilage condensation formation and distribution suggested that the core genetic network–cell behavioral mechanism that underlies this biological lattice gas might be sufficient to account for pattern formation in the limb cell micromass system and corresponding features of in vivo limb development. However, the model deviated from biological reality in several important ways. (1) Mesenchymal cells in vitro are initially surrounded by a small layer of ECM that separates them by less than a cell diameter. Those that undergo condensation round up, reducing their surface area, but do not move away from adjacent noncondensing cells. Therefore, unlike the situation in the model of Kiskowski et al. [26], mesenchymal condensation in micromass culture does not involve accumulation of cells at particular sites with concomitant depletion of cells in surrounding zones. (2) The representation of cells, morphogens, and ECM on a common grid is physically unrealistic. This is not simply a matter of pixel scale: molecular substances can indeed form deposits and gradients on the same linear scale as cells (∼10 μm), and a “molecular” pixel could be considered to correspond to thousands of molecules. Nonetheless, the dynamics of morphogen transport is continuous and is represented in an inauthentically saltatory fashion by pixel displacement on a grid of the same mesh size as that supporting cell translocation. (3) Whereas the model of Kiskowski et al. made the assumption that cells halt their motion when they encounter suprathreshold levels of extracellular fibronectin [26], this does not agree with measurements [31,32] indicating that cells actually slightly increase their speed of motion as they enter condensation centers and have a finite probability of escaping from these foci.

Despite the successes of the model of Kiskowski et al. [26], it was unknown whether removing its artifactual aspects and replacing them with more realistic assumptions would lead to similarly authentic results. We have therefore designed a more sophisticated model that overcomes each of the listed deficiencies of the earlier one. The cells in the new model are extended, multipixel objects that can change shape in the plane and “round up” by moving pixels into a virtual third dimension. The model cells are separated by less than a cell diameter, condense without denuding the regions surrounding condensation centers, and are not irreversibly trapped upon entering a center. Finally, two grids of different mesh size are used for cell and molecular dynamics.

We have found that not only does this improved model reproduce the experimental data accounted for by the model of Kiskowski et al., but that additional morphogenetic features of the micromass culture system are simulated as well. Moreover, potential dynamic properties of the developmental process not seen in the earlier simulations, and not capable of being distinguished on the basis of existing experimental data, were disclosed in simulations using the new model, which has therefore provided motivation for further empirical tests.

## Results

### Cell Representation

We represented each model cell as an extended object on a 2-D spatial grid. The rate and probability at which cells move (by random walk) and change shape are parameterized separately from movement of molecules so that they can be calibrated to the scale of actual biological cells. Each model cell behaves according to a predefined set of experimentally motivated rules involving morphogen dynamics controlling the production and deposition of fibronectin (see Materials and Methods).

**Figure 2:**
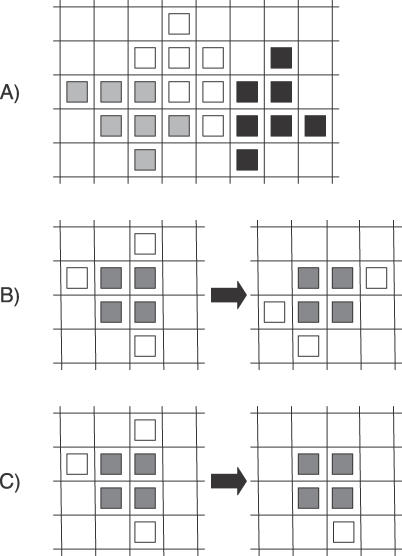
Multipixel Spatial Representation of Cells (A) Three cells on the spatial grid each occupying seven pixels. (B) Cell changes shape. The region of the cell that contains the nucleus, indicated by the four gray pixels, is structurally maintained; two border pixels move to new locations, and one border pixel (top right) displaces a nucleus pixel, which gets shifted to the right. (C) Cell rounding-up on fibronectin. The surface area in the presence of suprathreshold amounts of fibronectin is reduced with two border pixels moving into a quasi-third dimension above the cell.

We chose the simplest multipixel representation of limb mesenchymal cells subject to the following biological constraints: (1) cells have essentially isotropic geometry (i.e., they do not elongate in the direction of migration, but rather probe their environment by extending short randomly oriented projections); (2) the cell nucleus is also isotropic but is relatively unchanging in shape and comprises on the order of half the cell volume; and (3) cells in fibronectin-rich, condensing areas of the micromass round up such that their cross-section in the plane of the culture is significantly reduced [32]. Model cells (initially comprising seven pixels; Figure 2A) are therefore permitted to change shape consistent with maintaining four pixels in a two-by-two square (kernel) configuration that represents the portion of the cell that contains the nucleus (Figure 2B) (although one or more pixels of the central block can exchange with peripheral pixels at each time-step). Cells respond to suprathreshold levels of fibronectin by shrinking their area from seven pixels to five pixels (corresponding to rounding up into a virtual third dimension; Figure 2C) and increasing the rate at which they move and change shape. Once a cell ventures onto fibronectin it has the tendency to remain there, with a low probability of leaving the condensation. Seven pixels is the smallest multipixel representation that allows for both shape change events and appropriate cross-sectional area change when cells round up while maintaining a multipixel kernel. The relatively small cell-size representation in the model allows us to run extensive simulations with large numbers of cells for a wide range of different parameters. Smaller representations essentially reduce to a particle system; it is straightforward to add more pixels if greater cell shape fidelity is desired. (See Materials and Methods for implementation of all the above.)

### Experimental Constraints on Parameter Values

The degrees of freedom built into our model allowed us to calibrate some of the simulation parameters with experimentally determined values obtained in related or analogous systems. In particular, the diffusion rate of the activator morphogen and that of mesenchymal cells correspond well to experimental values, and they both play an important role in the resultant behavior of the model.

Lander et al. [33] calculated the effective diffusion coefficient for a molecule the size and shape of Dpp, a morphogen of the same superfamily as TGF-β, to be 10 μm^2^/s. We have used this value, although the actual value may differ due to varying capacities of members of the superfamily to bind to variable microenvironments [34]. In the present model, the diffusion rate for the activator morphogen in the reaction–diffusion system was found to be a key parameter for determining the size of the resultant patterns (see below). If the diffusion rate is too slow, the activator does not spread out across a sufficiently large area to produce broad condensations; in contrast, if the diffusion rate is too fast, the activator spreads out too much, thus preventing patterns from even forming.

The effective “diffusion” rate for cells is considerably slower than that of morphogens, and cells do not move significant distances over the time period of precartilage condensation formation [31,35,36]. (Diffusion of cells is of course not due to Brownian motion, as is molecular diffusion, but rather results from randomly directed cell locomotion based on internally generated surface protrusive forces.) Tracking of cells in time-lapse videos of developing chicken limb precartilage mesenchyme showed that they move with an average diffusion coefficient of ∼0.5 μm^2^/min in noncondensed regions and slightly faster in condensations [32]. We have therefore incorporated these experimental values into our model, increasing the cell diffusion rate by 50% in the presence of a threshold level of fibronectin (see Materials and Methods). In addition, we cause the area of cells situated on fibronectin to shrink in the presence of suprathreshold levels of fibronectin, corresponding to the observed rounding-up of cells in mesenchymal condensations.

Using the experimental values for activator and cell diffusion coefficients greatly facilitated choosing other parameters so that appropriately sized and spaced condensations formed in silico. This contrasted with parameter searches performed with nonbiological choices of activator and cell diffusion coefficients. In those cases, no realistic patterns formed in scores of simulations.

The inhibitor morphogen, elicited when cells in incipient condensations are exposed to one or more ectodermally produced fibroblast growth factors [30], must spread at a faster rate than the activator morphogen for stable patterns to be generated according to the reaction–diffusion dynamics. We performed a number of simulations that varied the ratio between the activator diffusion rate, which was kept constant, and the inhibitor diffusion rate. Consistent pattern formation was obtained when the inhibitor diffuses at a rate four to eight times faster than the activator. At a slower than 4-fold ratio, the patterns degraded in consistency until the point where no patterns were produced at all, which occurred when both diffusion rates were almost equal (Table 1). Beyond the 8-fold ratio, consistent patterns were still produced (results not shown). The relatively small ratio between the two diffusion rates makes the hypothesis of a diffusible inhibitor of condensation formation [30,37] biologically plausible.

**Table 1:**
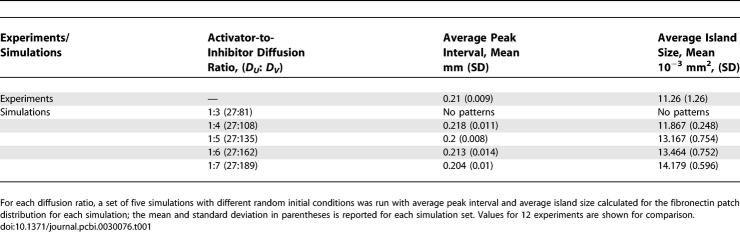
Variation of Average Peak Interval and Average Island Size over a Range of Diffusion Ratios

Experimental evidence indicated that limb mesenchymal cells in vitro respond to transient elevation in TGF-β concentration early during the culture period by upregulating fibronectin production for at least a day [2]. We have therefore assumed for the simulations described below that cells are induced to produce and secrete fibronectin by their first suprathreshold exposure to activator and become unresponsive to later exposures to activator. Furthermore, we have assumed that cells that are not exposed to inducing levels of activator during a critical period follow an alternative fibroblastic differentiation pathway [38], rendering them similarly unresponsive to later exposure to activator.

### Simulation of Condensation Patterns

**Figure 3:**
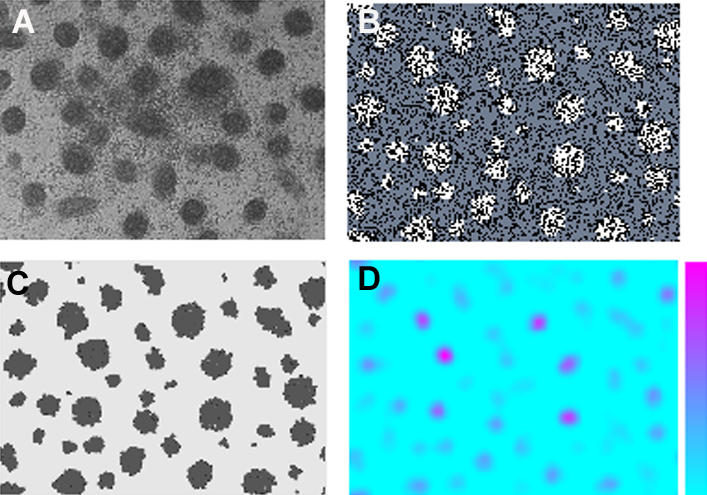
In Vitro and Oscillatory Regime Simulation Images for Spot-Like Precartilage Condensations (A) Discrete spot-like precartilage condensations that have formed after 72 h in a micromass culture of 5-d leg bud apical zone limb mesenchymal cells, visualized by Hoffman Contrast Modulation optics. Actual size of the microscopic field is 1 × 1.4 mm, and each condensation contains approximately 30–50 tightly packed cells. (B) Spatial grid of equal physical size to (A) containing over 6,000 cells produced by simulation using the parameter values in Table 2 showing clusters of fibronectin-producing differentiated cells (white), nondifferentiated cells (blue-gray), and empty space between cells (black). Each cluster contains on average ∼30 cells. (C) Spatial grid of same simulation as (B) showing fibronectin-rich patches (black) produced by the differentiated cells. (D) Spatial grid of same simulation as (B) showing activator concentrations at time slightly after the initial onset of cell differentiation. The color bar indicates the range, with magenta for high concentration and light blue for low concentration.

**Figure 4:**
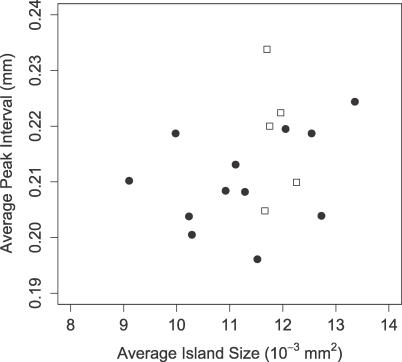
Average Peak Interval versus Average Island Size for Oscillatory Regime Averages are shown for 12 experimental (circle) and five simulation (square) points using parameter values in Table 2 with different random initial conditions. All simulations were run for 3,000 iterations with periodic boundary conditions.

Consistent with the experimental constraints described above, we searched for a parameter set in the model that reproduces the formation of precartilage condensation patterns. We calculated the average interval of the centroids (“peak interval”) [39] and the average island size of the fibronectin patches [26] for five simulation images and compared the values with those obtained from 12 in vitro condensation images such as that in Figure 3A. The results (Figure 4) indicate that our enhanced model reproduces the pattern of precartilage condensations equally as well as the model of Kiskowski et al. [26].

**Table 2:**
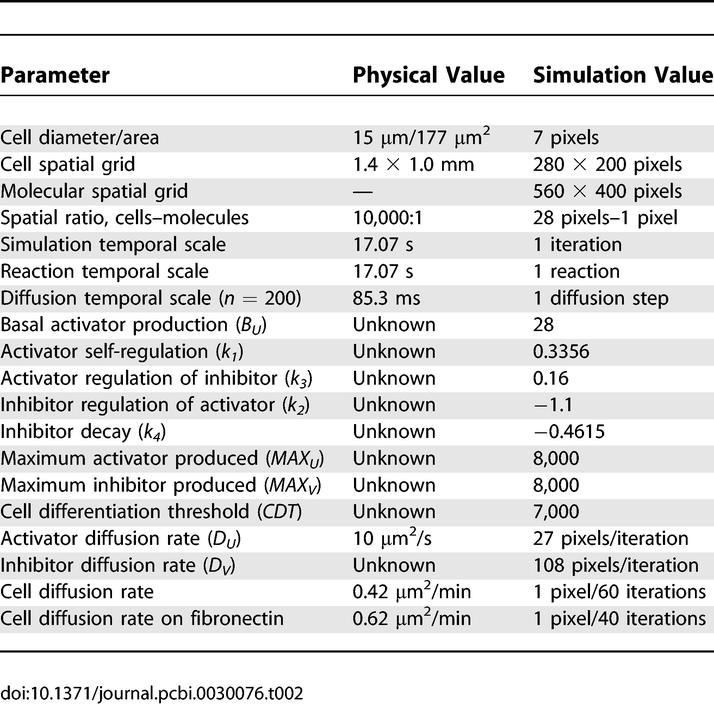
Calibrated Simulation Parameters to Known Physical Values

Different views of one simulation with parameters chosen within the “standard” range are shown in Figure 3B–3D. The distribution of condensations (Figure 3D) conforms very well to the photograph of the 72-h culture (Figure 3A), although the cells in the individual in silico condensations are not tightly packed as they are in the in vitro ones. This is not unexpected, since the model at present lacks representation of a cell–cell adhesion molecule, several of which are upregulated at condensation sites in limb mesenchyme [9,10]. The shape change of the model cells once they encounter fibronectin does nonetheless lead to a realistically higher cell density in condensed versus noncondensed regions of the simulated cultures.

The simulated distribution of fibronectin (Figure 3C) conforms to the distribution of condensations, as expected from immunolocalization studies [5]. The distribution of activator peaks at the time-point shown in Figure 3D maps out the set of eventual condensations. Previous experimental studies show TGF-β localization to anticipate the formation of condensations by up to a day [2], and to trigger the subsequent production of fibronectin after a brief, transient exposure [2]. The model, with different parameter choices, leads to realistic condensation patterns with either transient (as in the simulation shown in Figure 3B–3D) or stable activator patterns (see below).

**Figure 5:**
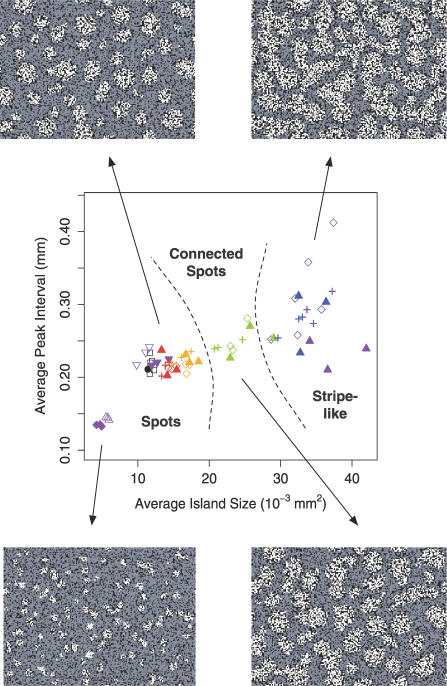
Variation in Some of the Key Parameters Induces Morphological Changes in the Resultant Spatial Patterns from Distinct Spots to Connected Spots to Stripe-Like Patterns Average peak interval versus average island size for variations in the some of the key parameters are shown: +5% (diamond) and −5% (filled diamond) for *k_1_*, +5% (triangle) and −5% (filled triangle) for *k_3_*, +5% (inverted triangle) and −5% (filled inverted triangle) for *k_2_*, and +5% (+) for *k_4_*. The colored points are a gradient of variations: 1% (red), 2% (orange), 3% (green), 4% (blue), and 5% (violet). Also shown are the five simulations (square) using the standard parameter values in Table 2 and the mean for the 12 experiments (circle). All simulations were run for 3,000 iterations with periodic boundary conditions.

We explored the robustness of the parameter set by varying key parameters independently (±5%); results can be seen in Figure 5. Minor variation of the inhibitor strength on activator (*k_2_*) by either +5% or −5% produced little change in the resulting condensation patterns. Instead, the temporal dynamics were modified, causing an increase and decrease in the period of the morphogen oscillations, respectively, with the +5% and −5% changes. For a decrease of 5% in the activator self-regulation (*k_1_*) or an increase of 5% in the activator regulation of inhibitor (*k_3_*), smaller condensations were produced with the condensations spaced further apart from one another. For a 5% increase in *k_1_* or a decrease of 5% in *k_3_*, condensation patterns greatly expanded in size such that the condensations touched one another, producing a pattern of interconnected stripes instead of spots. Similar results were also obtained if the inhibitor decay (*k_4_*) was increased by 5%. For a 5% decrease in *k_4_*, the chemical reaction was effectively damped, and no patterns were produced.

Consistent with observations of limb precartilage development in vitro and in vivo, our simulation results indicate that cells can form condensation patterns by undergoing small displacements of less than a cell diameter, packing more closely by changing their shapes, while maintaining a relatively uniform cell density across the entire spatial domain.

Given the possibility that choices of spatial domain and boundary conditions could lead to simulation artifacts, we sampled various alternatives in combination and investigated changes in the resulting condensation patterns.

With respect to the spatial domain, we ran simulations with rectangular grids of various widths and heights (unpublished data); this produced no noticeable effects on the size, shape, or distribution of the condensations. We conclude that the total area of the spatial domain determines only the number of condensations.

We also ran simulations with periodic and no-flux conditions. In periodic conditions, grid boundaries are connected together simulating a continuous space, whereas the no-flux boundary acts as a barrier. Both types of boundary conditions produced similar results for the size, shape, and distribution of the condensation patterns apart from the expected pattern truncations under no-flux conditions (unpublished data).

### Two Dynamic Regimes in Condensation Pattern Formation

**Figure 6:**
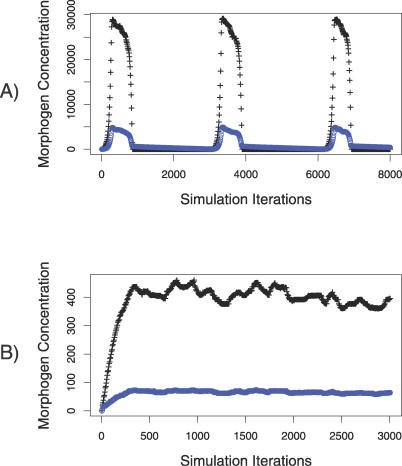
Dynamics of Oscillatory and Stationary Regimes (A) Oscillatory regime produces transient patterns that repeat over time but are spatially stochastic. (B) Stationary regime produces stable patterns with minor stochastic fluctuations around an equilibrium concentration. Graphs show the maximum concentration value for a single pixel across the entire molecular grid (that pixel lies within an activator peak as in Figure 3D but may shift from peak to peak as concentrations vary) for activator (black) and inhibitor (blue) morphogens.

Our simulations disclosed two regimes of behavior in the reaction–diffusion system of morphogens (Figure 6). In one regime, the maximum concentration levels for the two morphogens are characterized by a stationary value; this regime appears when the chemical reaction is slow (i.e., the production rate of the activator morphogen is balanced with the production rate of the inhibitor morphogen; Figure 6B). In the other regime, the concentrations levels for the two morphogens had an oscillatory behavior; concentrations increase up to a peak value, decrease back down to almost zero, and then continually repeat that cycle (Figure 6A). The oscillatory regime occurs when the chemical reaction is fast but a cap exists for the maximum amount of morphogen produced for a single reaction step.

Both regimes for the reaction–diffusion system can produce condensations patterns in the range of experimental values for size and distribution. (See Figures 3 and 4 for the oscillatory regime and Figures S1 and S2 for the stationary regime).

The limits on morphogen production (*MAX_U_* and *MAX_V_* in Table 2) induce the oscillatory regime by restricting production of activator while still allowing production of inhibitor, whose concentration has not yet reached the limit. The result is that inhibitor concentrations build up in the system; the inertia of inhibitor concentration dampens activator production throughout the whole system, which quickly accelerates and reduces the activator concentration down to basal levels. Cells continue to produce a basal amount of activator, so over time conditions are reproduced for the onset of morphogen pattern formation. The dynamics repeat, with transient patterns being formed, though the spatial arrangement of the peaks varies unpredictably from one oscillation to the next.

Variations in the limits on morphogen production in the oscillatory regime produced minimal changes in the average peak interval and average island size of the fibronectin patch distribution (Table 3). The oscillatory regime is more robust for higher limits and breaks down when the concentrations are low. In contrast, the stationary regime operates in the lower concentration levels of the morphogens.

**Table 3:**
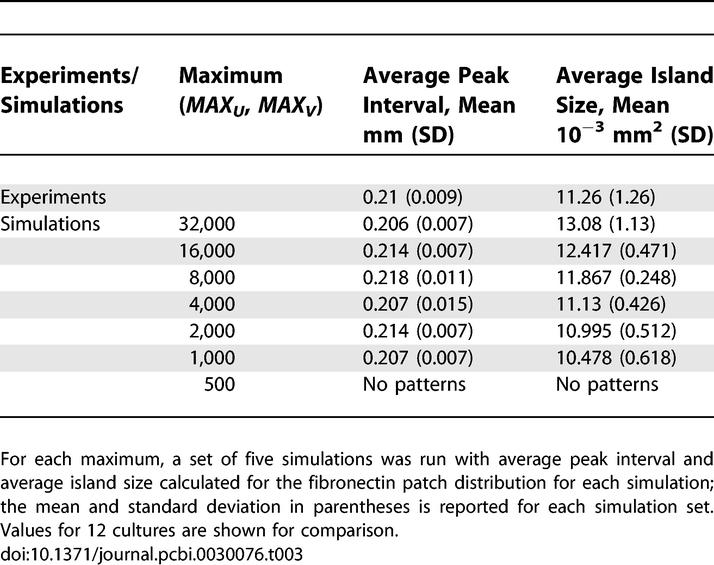
Robustness of Average Peak Interval and Average Island Size over a Range of Production Maximums

The oscillatory regime is robust to a noisy threshold level for cell differentiation. Simulations where each cell's threshold is randomly assigned from a normal distribution, *N* (9,000; 1,000), instead of a constant value, produce only slight variation in the average peak interval and average island size despite the large deviation in the threshold levels. However, the stationary regime is sensitive to the threshold level for cell differentiation as a modest variation, *N* (2,400; 170), completely disrupts the spatial distribution of the fibronectin patches (unpublished data).

The formation of patterns in the stationary regime is sensitive to the period that cells are exposed to activator morphogen and to the threshold level for cell differentiation. If the exposure time is too short, small, irregularly spaced condensations are produced. If the exposure is too long, irregularly shaped condensations are produced. Although the stationary regime produces stable activator peaks, those peaks tend to wander spatially over time due to the underlying cell diffusion. The oscillatory regime is less sensitive to the threshold level for cell differentiation, and a single transient pulse provides a well-defined exposure period.

### Formation of Stripe Patterns

**Figure 7:**
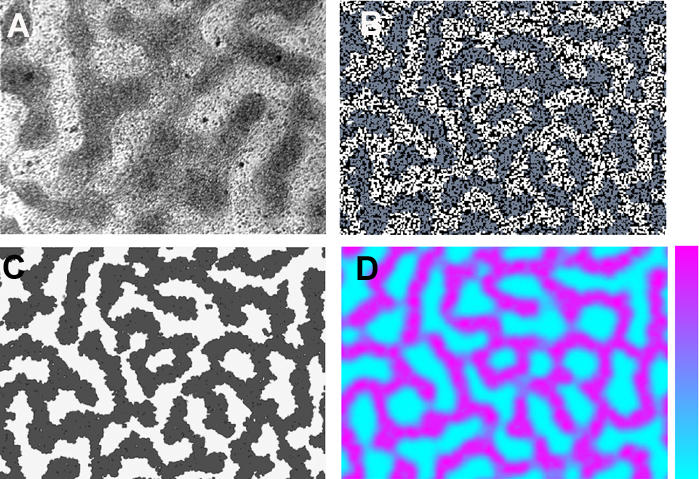
In Vitro and Simulation Images for Stripe-Like Precartilage Condensations (A) Stripe-like precartilage condensations. (B) Spatial grid containing more than 6,000 cells produced by simulation showing stripes of fibronectin-producing differentiated cells (white), nondifferentiated cells (blue-gray), and empty space between cells (black). (C) Spatial grid of same simulation as (B) showing fibronectin-rich stripes (black) produced by the differentiated cells. (D) Spatial grid of same simulation as (B) showing activator concentrations at time slightly after the initial onset of cell differentiation. The color bar indicates the range, with magenta for high concentration and light blue for low concentration.

While the focus of our model has been on producing the spot patterns typically seen in leg-cell cultures [5,40], the exact same model can produce stripe patterns with a slight adjustment to parameters (Figure 7). This is significant because uncontrolled variations in the preparation of cultures grown under the same conditions as the spot-producing ones occasionally give rise to stripe patterns (Figure 7A). When the reaction–diffusion system progresses to spot patterns, it goes through a brief period of partial stripe formation until dominant activator peaks stabilize the system into spot patterns. Reducing the limits on morphogen production (*MAX_U_* and *MAX_V_* in Table S1) prevents peaks of activator morphogen from dominating, and stable stripe patterns are maintained. This corresponds to theoretical analysis by Shoji and coworkers of reaction–diffusion systems with linear kinetics and constant constraints [41]; they show that stripe patterns are generated instead of spot patterns if the upper and lower constraints are equal distances from the equilibrium. Similar to the formation of spot patterns in the stationary regime, the formation of stripe patterns is sensitive to the duration of the period in which cells are exposed to activator morphogen.

## Discussion

We have demonstrated that parameter choices can be found for our quasi–3-D discrete model that reproduce the experimental distribution and size range of precartilage condensations in experimental micromass cultures. The performance of the model was equal to that of Kiskowski et al. [26], despite the imposition of realistic scaling and experimentally determined constraints.

The new model has allowed us to study the interplay between reaction–diffusion processes, fibronectin production, and cell–fibronectin interaction in greater detail than previously possible. In particular, our simulations disclosed two regimes in the interplay of the reaction–diffusion system of morphogens with fibronectin production and cell behavior. In one regime, stationary morphogen patterns were produced, followed by cell rearrangement into patterns of condensation. In the second regime, morphogen patterns were transient and oscillatory in time, and the induced fibronectin production (and consequent cell rearrangement) occurred with a delay. In addition, the dynamic characteristics of the second regime provide a natural explanation for apparent oscillatory effects of limb precartilage cell responses to TGF-β seen in previous experimental studies [2]. As mentioned in Results, the transient regime also exhibits less sensitivity than the stationary regime to several key system parameters, giving it plausibility as the more robust pattern-forming mechanism. However, in order to suppress “second-generation” condensation patterns due to the recurrence of activator peaks in this regime, we assumed that cell differentiation to a morphogen-nonresponsive state occurs rapidly relative to the period of oscillation. This assumption is obviously not needed for simulations in the stationary case; indeed, stable pattern formation in this regime would be consistent with extended (i.e., over a period of a day or more) susceptibility to perturbation by exogenous TGF-β. We are currently performing in vitro experiments analogous to earlier studies on the first day of development [2] to test this predicted difference, as well as some others.

Our model generates realistic patterns of precartilage condensation in high-density culture without the need to postulate direct cell–cell adhesive interactions. This feature appears to reflect biological reality. First, although the separation of condensing from noncondensing cells superficially resembles sorting out by differential adhesion (see [42] for a recent model of the latter process based on a free-energy minimization principle), haptotactic binding to fibronectin is sufficient to recruit limb precartilage mesenchymal cells, or even inert particles, into condensations [6]. Second, while as mentioned above, several cell–cell adhesive proteins, including N-CAM [9] and N-cadherin [10], are expressed at sites of condensation, their loss does not impair condensation-dependent skeletogenesis [43,44].

We note that in both the oscillatory and stationary cases, the region of parameter space that leads to realistic fibronectin patch and condensation patterns corresponds to activator morphogen peaks that are on the spatial scale of the condensations themselves. For the oscillatory regime, a small number of those peaks (see Videos S1 and S2) have relatively high and possibly nonphysiological activator and inhibitor concentrations (assuming morphogen units represent one or more protein molecules). If morphogen dynamics in these cultures is indeed oscillatory [2], this may represent an inauthentic aspect of our model, resulting from the use of the classic diffusion-dependent Turing-type morphogen scheme. We are therefore exploring alternative embodiments of the model using juxtacrine signaling, the role of which is suggested by recent demonstration of involvement of the Notch signaling pathway in the inhibitory branch of the condensation-patterning network [45]. Recent analyses have suggested that introducing juxtacrine signaling into the dynamics can bring reaction–diffusion pattern-forming systems that are otherwise biochemically implausible into more realistic parameter domains [46]. We note that our multipixel representation will enable the incorporation of cell asymmetry and polarity (a known feature of limb mesenchymal cells [47]) in future models using cell relay mechanisms.

The capacity of our model to generate both spots and stripes of precartilage condensation under slightly different parameter choices corresponds well to experimental results in which either morphotype may be generated under similar initial conditions. Because the developing limb itself generates its skeleton in the form of spots and stripes of precartilage condensation (Figure 1; see also [1]), this result of our simulations supports the applicability of the core molecular–genetic mechanism we have used to the understanding of both in vitro and in vivo chondrogenic pattern formation. Moreover, the flexibility and generality of the framework presented here makes it suitable for representing and testing other experimentally motivated models for periodic patterning in which cell movement and shape change is involved, such as the formation of feathers and hair [25,48] and teeth [49,50].

## Materials and Methods

### Mesenchymal cell cultures.

Cell cultures were prepared using precartilage mesenchymal tissue isolated from the myoblast-free distal 0.3 mm [51] of Hamburger and Hamilton stages 24–25 [52] leg buds of 5-d White Leghorn embryos (Moyer's Chicks, http://www.moyerschicks.com) under the conditions described for the standard cultures in Kiskowski et al. [26] (1.75 × 10^5^ cell per 10-μl spot in serum-free defined medium [53]). Living cultures were photographed using Hoffman Modulation Contrast optics (4× objective lens; Modulation Optics, Inc., http://www.modulationoptics.com) with condenser and polarizer adjusted to visualize cell condensations [7].

### Computational model and cell dynamics.

The spatial environment that cells and molecules occupy is modeled on a 2-D plane. The implementation provides support for multiple superimposed discrete grids of various spatial scales. In our current model, we use two scales: one for the cellular level and another finer-resolution scale for the molecular level. The coarsest resolution spatial scale is considered to be the base spatial scale, which is the cellular level for our model; all other grids are an integer ratio size of that base grid. The base spatial grid can be defined as a square or rectangular grid of any height and width, and all of the grids overlay one another and cover the same physical area.

Each cell is represented as a set of seven contiguous pixels operating on the base spatial grid as shown in Figure 2A. We maintained four pixels in a two-by-two square (kernel) configuration that represents the portion of the cell that contains the nucleus and allowed the remaining pixels to occupy the border region around the nucleus. Cells that round up shrink their spatial extent to five pixels (Figure 2C).

Cell diffusion was implemented as a random walk. If the cell moves, then all of its seven (or five) pixels move one pixel in the appropriate direction (up, down, left, right). Cells can also fluctuate in shape, yet such fluctuations maintain a structural representation of the central region containing the nucleus by preserving intact a two-by-two square block of pixels. Therefore, shape fluctuations are restricted to the motion of the three (or one) border pixels around the nucleus which either move to new border pixels or displace nucleus pixels; Figure 2B gives an example of both types of fluctuations for a cell changing shape. Cells are prevented from overlapping each other when they move or change shape.

### Morphogen reaction–diffusion dynamics.

In our discrete representation of the Turing mechanism, a discrete number of activator and inhibitor molecules occupy each pixel on the grid, and each molecule is considered to have a spatial representation of just one pixel. We modeled the reaction dynamics of the activator and inhibitor molecules at each pixel as follows: let *U_t_* and *V_t_* be the concentration of the activator and inhibitor, respectively, at time *t*, and let *φ_t_* be an indicator function for the existence of a cell (*φ_t_* = 1 if cell is at the pixel; otherwise, *φ_t_* = 0) at time *t*.

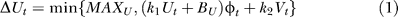


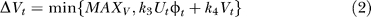



Equation 1 shows the change over time for each pixel on the grid of the activator morphogen concentration based upon a proportion (as defined by chemical reaction rates) of the current activator and inhibitor concentrations. Equation 2 shows the corresponding change over time for each pixel on the grid of the inhibitor morphogen. The activator morphogen is considered a positive self-regulating molecule and a positive regulator of the inhibitor; thus, chemical rate parameters *k_1_* and *k_3_* both have positive values. The inhibitor morphogen is considered a negative regulator of activator that decays over time; thus, chemical rate parameters *k_2_* and *k_4_* are both negative values.

In our model, production of the activator and inhibitor molecules, as represented by the parameters *k_1_* and *k_3_*, can only occur in the presence of a cell as enforced by *φ_t_*; however, the decay of activator and inhibitor, as represented by the parameters *k_2_* and *k_4_*, is considered to occur independent of cell presence. Cells are initially randomly distributed on the grid and secrete a small basal amount (*B_U_*) of activator morphogen that provides the initial concentration of activator; cells continue this basal production throughout the simulation, and inhibitor concentration starts at zero.

In keeping with the biology, we considered cells to respond to low concentrations of morphogens and thus represent morphogen molecules as discrete entities. Consequently, the morphogen concentrations (*U_t_*, *V_t_*) are whole numbers, and changes in the concentrations at a time-step are rounded to the nearest integer and prevented from becoming negative. Nonetheless, we treated the chemical rate parameters (*k_1_*, *k_2_*, *k_3_*, *k_4_*) for the two morphogens as averages of the reaction rates and allowed them to assume real number values.

In any physicochemical reaction there is limitation on how much reagent a single cell can realistically produce during any period of time. For this reason, our model provides separate parameters (*MAX_U_*, *MAX_V_*) for the maximum amount of activator and inhibitor that can be produced during a single reaction step. The maximums are imposed on individual pixels of the molecular grid rather than across the entire cell, consistent with polarization of limb mesenchymal cells [47]. This allows for small morphogen gradients to be present across the spatial extent of an individual cell through the spatially polarized secretion of morphogens. The peaks of activator concentration produced by the reaction–diffusion dynamics define a large-scale prepattern, equal in spatial area to the fibronectin patches, containing around 30 cells within a single patch. Polarization plays a role for the cells on the border region of the patch, whereas cells in the patch interior perceive a relatively constant morphogen concentration across their entire spatial extent.

Molecular diffusion from any pixel can occur randomly toward any of the four neighboring pixels (up, down, left, right). The diffusion rates (*D_U_*, *D_V_*) are scaled into a probability factor 0 *< p <* 1 and a time-step *n* such that *D = pn*. The probability determines the chance that a molecule will diffuse, and the time-step indicates how many opportunities a molecule has to diffuse for a single simulation iteration; if the molecule diffuses, then one of the four neighboring pixels is picked with equal probability. The chemical reaction operates at a much slower rate than molecular diffusion, so the time scales are separated with diffusion calculated at a small time-step and the reaction calculated at a longer time-step.

### Fibronectin production.

Fibronectin is a nondiffusing ECM molecule that forms the template for precartilage condensations. As the concentration levels of the activator morphogen increases in the presence of a cell, that cell produces fibronectin mRNA, which can then be translated into actual fibronectin protein molecules. The model supports a simple threshold level (*CDT*) such that once the sum of activator concentration across the entire spatial area of a cell exceeds that threshold value, the cell differentiates into a fibronectin-producing cell. Because we did not directly describe the level of fibronectin mRNA within the cell, the trigger for cell differentiation is separated from the actual production of fibronectin, and a model parameter defines the delay between cell differentiation and secretion of fibronectin.

We assumed that there is a critical period during which exposure, or lack of exposure, of cells to activator morphogen, causes them to either differentiate into fibronectin-producing cells or follow an alternative differentiation pathway. For purposes of simulation, we disabled the reaction–diffusion dynamics after this critical period (adjustable by a parameter) and prevented additional cells from differentiating. For the oscillatory regime, a single transient pulse (Figure 6B) defines the exposure period; reaction–diffusion dynamics are disabled when the activator morphogen returns to basal concentration levels. For the stationary regime, reaction–diffusion dynamics are disabled after 500 simulation iterations (see Figures S1 and S2).

When a cell produces fibronectin, a single unit representing a multimolecular complex is secreted with random probability for each of the pixels on the molecular grid in the cell's spatial domain, and each unit is allowed to perform an initial small diffusion of at most one pixel [26]. Production of fibronectin units continues until a maximum concentration level is reached at a pixel, although cells may still continue to produce fibronectin on pixels that have not yet reached the maximum. The production rate of fibronectin, the duration of such production, and the maximum amount of fibronectin allowed per pixel can be adjusted with model parameters.

### Model calibration.

In attempting to calibrate our model parameters with known empirical parameters, we wanted to correlate the spatial and temporal patterns produced by computer simulation results with in vivo and in vitro experiments. For spatial patterns, we considered the size, shape, and distribution of the fibronectin-rich spatial domains; for temporal patterns, we considered the reaction rates of activator and inhibitor production, the diffusion rates of both cells and molecules, the onset of fibronectin production, the production rate of fibronectin, and the shape and movement fluctuations of cells on fibronectin. The actual values for the set of key parameters used in the simulation and their corresponding physical measurements, if known, are shown in Table 2.

### Software implementation.

Whereas the previous model of Kiskowski et al. [26] was written in Matlab (http://www.mathworks.com), we rewrote the current model in the C programming language for efficiency, and then migrated it to the Objective-C programming language to take advantage of object-oriented features. We still used Matlab for visualizing data produced by simulation runs. The original source code is available as Dataset S1 accompanying this article, and can be obtained directly from the authors. We intend to continue developing the software by expanding the capability to add molecular and cellular detail to models of the limb micromass culture system and allied 2-D and quasi–3-D developmental systems.

## Supporting Information

Dataset S1 Source Code and Supporting Files for the Model (330 KB TAR)

Figure S1 In Vitro and Stationary Regime Simulation Images for Spot-Like Precartilage Condensations (1.0 MB TIF)

Figure S2 Average Peak Interval versus Average Island Size for Stationary Regime (4 KB PDF)

Table S1 Simulation Parameters (28 KB DOC)

Video S1 Activator Concentrations for Oscillatory Regime (3.8 MB MOV)

Video S2 Inhibitor Concentrations for Oscillatory Regime (3.4 MB MOV)

Video S3 Activator Concentrations for Stationary Regime (11.1 MB MOV)

Video S4 Inhibitor Concentrations for Stationary Regime (9.8 MB MOV)

## Abbreviations

ECM: extracellular matrix

## References

[pcbi-0030076-b001] Newman SA, Müller GB (2005). Origination and innovation in the vertebrate limb skeleton: An epigenetic perspective. J Exp Zoolog B Mol Dev Evol.

[pcbi-0030076-b002] Leonard CM, Fuld HM, Frenz DA, Downie SA, Massagué J (1991). Role of transforming growth factor-beta in chondrogenic pattern formation in the embryonic limb: Stimulation of mesenchymal condensation and fibronectin gene expression by exogenous TGF-β and evidence for endogenous TGF-β–like activity. Dev Biol.

[pcbi-0030076-b003] Miura T, Shiota K (2000). TGF β2 acts as an “activator” molecule in reaction–diffusion model and is involved in cell sorting phenomenon in mouse limb micromass culture. Dev Dyn.

[pcbi-0030076-b004] Chimal-Monroy J, Rodriguez-Leon J, Montero JA, Gañan Y, Macias D (2003). Analysis of the molecular cascade responsible for mesodermal limb chondrogenesis: *sox* genes and BMP signaling. Dev Biol.

[pcbi-0030076-b005] Downie SA, Newman SA (1995). Different roles for fibronectin in the generation of fore and hind limb precartilage condensations. Dev Biol.

[pcbi-0030076-b006] Frenz DA, Akiyama SK, Paulsen DF, Newman SA (1989). Latex beads as probes of cell surface–extracellular matrix interactions during chondrogenesis: Evidence for a role for amino-terminal heparin-binding domain of fibronectin. Dev Biol.

[pcbi-0030076-b007] Frenz DA, Jaikaria NS, Newman SA (1989). The mechanism of precartilage mesenchymal condensation: A major role for interaction of the cell surface with the amino-terminal heparin-binding domain of fibronectin. Dev Biol.

[pcbi-0030076-b008] Gehris AL, Stringa E, Spina J, Desmond ME, Tuan RS (1997). The region encoded by the alternatively spliced exon IIIA in mesenchymal fibronectin appears essential for chondrogenesis at the level of cellular condensation. Dev Biol.

[pcbi-0030076-b009] Widelitz RB, Jiang TX, Murray BA, Chuong CM (1993). Adhesion molecules in skeletogenesis: II. Neural cell adhesion molecules mediate precartilaginous mesenchymal condensations and enhance chondrogenesis. J Cell Physiol.

[pcbi-0030076-b010] Oberlender SA, Tuan RS (1994). Expression and functional involvement of N-cadherin in embryonic limb chondrogenesis. Development.

[pcbi-0030076-b011] Newman SA, Tomasek JJ, Comper WD (1996). Morphogenesis of connective tissues. Extracellular matrix.

[pcbi-0030076-b012] Hall BK, Miyake T (1995). Divide, accumulate, differentiate: Cell condensation in skeletal development revisited. Int J Dev Biol.

[pcbi-0030076-b013] Hall BK, Miyake T (2000). All for one and one for all: Condensations and the initiation of skeletal development. Bioessays.

[pcbi-0030076-b014] Maini PK (1989). Spatial and spatio-temporal patterns in a cell-haptotaxis model. J Math Biol.

[pcbi-0030076-b015] Dillon R, Maini PK, Othmer HG (1994). Pattern formation in generalized Turing systems. J Math Biol.

[pcbi-0030076-b016] Maini PK, Benson DL, Sherratt JA (1992). Pattern formation in reaction–diffusion models with spatially inhomogeneous diffusion coefficients. Math Med Biol.

[pcbi-0030076-b017] Orme ME, Chaplain MAJ (1996). A mathematical model of the first steps of tumour-related angiogenesis: Capillary sprout formation and secondary branching. Math Med Biol.

[pcbi-0030076-b018] Anderson ARA, Chaplain MAJ (1998). Continuous and discrete mathematical models of tumour-induced angiogenesis. Bull Math Biol.

[pcbi-0030076-b019] Marée AF, Hogeweg P (2001). How amoeboids self-organize into a fruiting body: Multicellular coordination in Dictyostelium discoideum. Proc Natl Acad Sci U S A.

[pcbi-0030076-b020] Maree AF, Hogeweg P (2002). Modelling Dictyostelium discoideum morphogenesis: The culmination. Bull Math Biol.

[pcbi-0030076-b021] Alber M, Kiskowski M, Jiang Y, Newman S, Dangelmayr G, Oprea I (2004). Biological lattice gas models. Dynamics and bifurcation of patterns in dissipative systems.

[pcbi-0030076-b022] Sozinova O, Jiang Y, Kaiser D, Alber M (2005). A three-dimensional model of myxobacterial aggregation by contact-mediated interactions. Proc Natl Acad Sci U S A.

[pcbi-0030076-b023] Sozinova O, Jiang Y, Kaiser D, Alber M (2006). A three-dimensional model of fruiting body formation. Proc Natl Acad Sci U S A.

[pcbi-0030076-b024] Merks RM, Brodsky SV, Goligorksy MS, Newman SA, Glazier JA (2006). Cell elongation is key to in silico replication of in vitro vasculogenesis and subsequent remodeling. Dev Biol.

[pcbi-0030076-b025] Jiang TX, Widelitz RB, Shen WM, Will P, Wu DY (2004). Integument pattern formation involves genetic and epigenetic controls: Feather arrays simulated by digital hormone models. Int J Dev Biol.

[pcbi-0030076-b026] Kiskowski MA, Alber MS, Thomas GL, Glazier JA, Bronstein NB (2004). Interplay between activator-inhibitor coupling and cell-matrix adhesion in a cellular automaton model for chondrogenic patterning. Dev Biol.

[pcbi-0030076-b027] Chaturvedi R, Huang C, Kazmierczak B, Schneider T, Izaguirre JA (2005). On multiscale approaches to three-dimensional modelling of morphogenesis. J R Soc Interface.

[pcbi-0030076-b028] Christley S, Newman SA, Alber MS, Deutsch IA, Brusch L, Byrne H, de Vries G, Herzel HP (2007). Agent-based model for developmental pattern formation with multiscale dynamics and varying cell geometry. Mathematical modeling of biological systems, volume I.

[pcbi-0030076-b029] Christley S, Newman SA, Alber MS, Rocha LM, Yaeger LS, Bedau MA, Floreano D, Goldstone RL, Vespignani A (2006). Modeling of pattern formation in cell cultures. Artificial life X: proceedings of the tenth international conference on the simulation and synthesis of living systems.

[pcbi-0030076-b030] Moftah MZ, Downie SA, Bronstein NB, Mezentseva N, Pu J (2002). Ectodermal FGFs induce perinodular inhibition of limb chondrogenesis in vitro and in vivo via FGF receptor 2. Dev Biol.

[pcbi-0030076-b031] Ede DA, Flint OP, Wilby OK, Colquhoun P, Ede DA, Hinchliffe JR, Balls M (1977). The development of precartilage condensations in limb bud mesenchyme in vivo and in vitro. Vertebrate limb and somite morphogenesis.

[pcbi-0030076-b032] Cui C (2005). Dynamics of cell movement and tissue motion in gastrulation and micromass cell culture.

[pcbi-0030076-b033] Lander AD, Nie Q, Wan FY (2002). Do morphogen gradients arise by diffusion?. Dev Cell.

[pcbi-0030076-b034] Ohkawara B, Iemura S, ten Dijke P, Ueno N (2002). Action range of BMP is defined by its N-terminal basic amino acid core. Curr Biol.

[pcbi-0030076-b035] Gould RP, Day A, Wolpert L (1972). Mesenchymal condensation and cell contact in early morphogenesis of the chick limb. Exp Cell Res.

[pcbi-0030076-b036] Thorogood PV, Hinchliffe JR (1975). An analysis of the condensation process during chondrogenesis in the embryonic hind limb. J Embryol Exp Morphol.

[pcbi-0030076-b037] Miura T, Maini PK (2004). Speed of pattern appearance in reaction–diffusion models: Implications in the pattern formation of limb bud mesenchyme cells. Bull Math Biol.

[pcbi-0030076-b038] Newman SA (1980). Fibroblast progenitor cells of the embryonic chick limb. J Embryol Exp Morphol.

[pcbi-0030076-b039] Miura T, Komori M, Shiota K (2000). A novel method for analysis of the periodicity of chondrogenic patterns in limb bud cell culture: Correlation of in vitro pattern formation with theoretical models. Anat Embryol (Berl).

[pcbi-0030076-b040] Downie SA, Newman SA (1994). Morphogenetic differences between fore and hind limb precartilage mesenchyme: Relation to mechanisms of skeletal pattern formation. Dev Biol.

[pcbi-0030076-b041] Shoji H, Iwasa Y, Kondo S (2003). Stripes, spots, or reversed spots in two-dimensional Turing systems. J Theor Biol.

[pcbi-0030076-b042] Kafer J, Hogeweg P, Maree AFM (2006). Moving forward moving backward: Directional sorting of chemotactic cells due to size and adhesion differences. PLoS Comput Biol.

[pcbi-0030076-b043] Cremer H, Lange R, Christoph A, Plomann M, Vopper G (1994). Inactivation of the *N-CAM* gene in mice results in size reduction of the olfactory bulb and deficits in spatial learning. Nature.

[pcbi-0030076-b044] Luo Y, Kostetskii I, Radice GL (2005). N-cadherin is not essential for limb mesenchymal chondrogenesis. Dev Dyn.

[pcbi-0030076-b045] Fujimaki R, Toyama Y, Hozumi N, Tezuka K (2006). Involvement of Notch signaling in initiation of prechondrogenic condensation and nodule formation in limb bud micromass cultures. J Bone Miner Metab.

[pcbi-0030076-b046] Rauch EM, Millonas MM (2004). The role of *trans*-membrane signal transduction in Turing-type cellular pattern formation. J Theor Biol.

[pcbi-0030076-b047] Holmes LB, Trelstad RL (1980). Cell polarity in precartilage mouse limb mesenchyme cells. Dev Biol.

[pcbi-0030076-b048] Widelitz RB, Baker RE, Plikus M, Lin CM, Maini PK (2006). Distinct mechanisms underlie pattern formation in the skin and skin appendages. Birth Defects Res C Embryo Today.

[pcbi-0030076-b049] Thesleff I, Keranen S, Jernvall J (2001). Enamel knots as signaling centers linking tooth morphogenesis and odontoblast differentiation. Adv Dent Res.

[pcbi-0030076-b050] Salazar-Ciudad I, Jernvall J (2002). A gene network model accounting for development and evolution of mammalian teeth. Proc Natl Acad Sci U S A.

[pcbi-0030076-b051] Brand B, Christ B, Jacob HJ (1985). An experimental analysis of the developmental capacities of distal parts of avian leg buds. Am J Anat.

[pcbi-0030076-b052] Hamburger V, Hamilton HL (1951). A series of normal stages in the development of the chick embryo. J Morphol.

[pcbi-0030076-b053] Paulsen DF, Solursh M (1988). Microtiter micromass cultures of limb-bud mesenchymal cells. In Vitro Cell Dev Biol.

[pcbi-0030076-b054] Forgacs G, Newman SA (2005). Biological physics of the developing embryo.

