# Supplementary figures and images for: Patterns of Mesenchymal Condensation in a Multiscale, Discrete Stochastic Model
Source: PLoS Comput Biol. 2007 Apr 27;3(4):e76. doi: 10.1371/journal.pcbi.0030076 (PMC1857812; doi:10.1371/journal.pcbi.0030076)

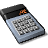

Supplement: Dataset S1 — (330 KB TAR) [file pcbi.0030076.sd001.tar › limb/LimbChondrogenesis.app.tiff]

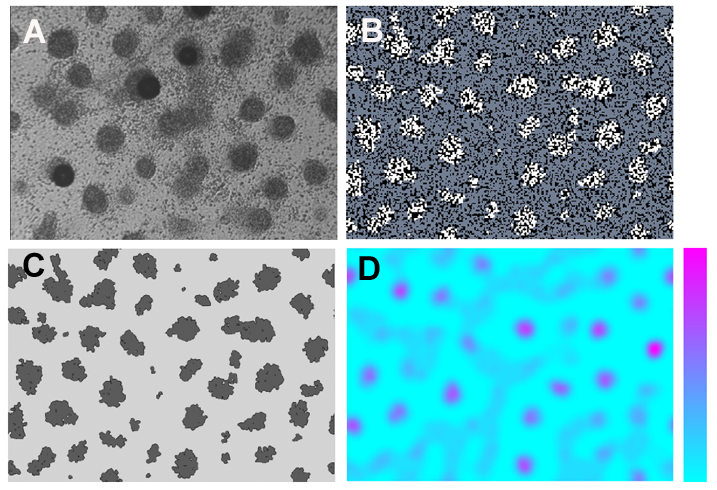

Supplement: Figure S1 — (1.0 MB TIF) [file pcbi.0030076.sg001.tif]

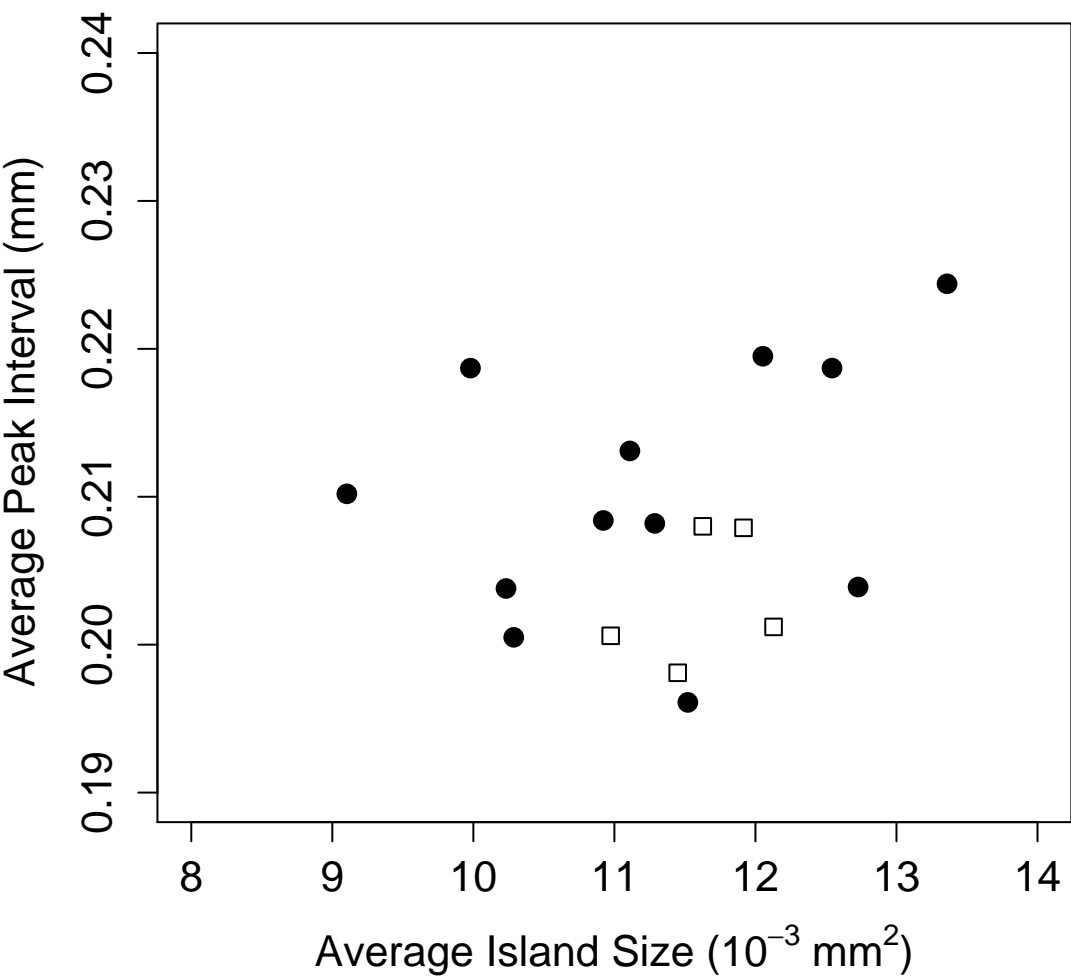

Supplement: Figure S2 — (4 KB PDF) [file pcbi.0030076.sg002.pdf]
