# Supplementary material for: Patterns of Mesenchymal Condensation in a Multiscale, Discrete Stochastic Model
Source: PLoS Comput Biol. 2007 Apr 27;3(4):e76. doi: 10.1371/journal.pcbi.0030076 (PMC1857812; doi:10.1371/journal.pcbi.0030076)
Supplement: Table S1 — (28 KB DOC) [file pcbi.0030076.st001.doc]

Table S1. Simulation Parameters

| **Parameter** | **Oscillatory** | **Stationary** | **Stripes** |
| --- | --- | --- | --- |
| Cell diameter/area | 7 pixels | 7 pixels | 7 pixels |
| Cell spatial grid | 280 × 200 pixels | 280 × 200 pixels | 280 × 200 pixels |
| Molecular spatial grid | 560 × 400 pixels | 560 × 400 pixels | 560 × 400 pixels |
| Spatial ratio cells : molecules | 28 pixels : 1 pixel | 28 pixels : 1 pixel | 28 pixels : 1 pixel |
| Basal activator production (*B_U_*) | 28 | 28 | 28 |
| Activator self-regulation (*k_1_*) | 0.3356 | 0.3146 | 0.3356 |
| Activator regulation of inhibitor (*k_3_*) | 0.16 | 0.1584 | 0.16 |
| Inhibitor regulation of activator (*k_2_*) | -1.1 | -1.1 | -1.1 |
| Inhibitor decay (*k_4_*) | -0.4615 | -0.4615 | -0.4615 |
| Maximum activator produced (*MAX_U_*) | 8000 | 8000 | 35 |
| Maximum inhibitor produced (*MAX_V_*) | 8000 | 8000 | 35 |
| Cell differentiation threshold (*CDT*) | 7000 | 2400 | 2200 |
| Activator diffusion rate (*D_U_*) | 27 pixels/iteration | 30 pixels/iteration | 27 pixels/iteration |
| Inhibitor diffusion rate (*D_V_*) | 108 pixels/iteration | 120 pixels/iteration | 108 pixels/iteration |
| Cell diffusion rate | 1 pixel/60 iterations | 1 pixel/60 iterations | 1 pixel/60 iterations |
| Cell diffusion rate on fibronectin | 1 pixel/40 iterations | 1 pixel/40 iterations | 1 pixel/40 iterations |
